# Photoacoustic in vivo 3D imaging of tumor using a highly tumor-targeting probe under high-threshold conditions

**DOI:** 10.1038/s41598-020-76281-1

**Published:** 2020-11-09

**Authors:** Hisatsugu Yamada, Natsuki Matsumoto, Takanori Komaki, Hiroaki Konishi, Yu Kimura, Aoi Son, Hirohiko Imai, Tetsuya Matsuda, Yasuhiro Aoyama, Teruyuki Kondo

**Affiliations:** 1 Department of Energy and Hydrocarbon Chemistry, Graduate School of Engineering, Kyoto University, Katsura, Nishikyo-ku, Kyoto, 615-8510 Japan; 2 Department of Systems Science, Graduate School of Informatics, Kyoto University, Yoshida-honmachi, Sakyo-ku, Kyoto, 606-8501 Japan; 3 Present Address: Graduate School of Technology, Industrial and Social Sciences, Tokushima University, Tokushima, Japan

**Keywords:** Cancer imaging, Near-infrared spectroscopy, Imaging, Sensors and probes, Fluorescent dyes, Diagnosis, Medical imaging, Diagnostics, Drug delivery, Target identification, Cancer imaging

## Abstract

Three-dimensional (3D) representation of a tumor with respect to its size, shape, location, and boundaries is still a challenge in photoacoustic (PA) imaging using artificial contrast agents as probes. We carried out PA imaging of tumors in mice using 800RS-PMPC, which was obtained by coupling of 800RS, a near-infrared cyanine dye, with PMPC, a highly selective tumor-targeting methacrylate polymer having phosphorylcholine side chains, as a probe. The conjugate 800RS-PMPC forms compact nanoparticles (*d*_DLS_ = 14.3 nm), retains the biocompatibility of the parent polymer (PMPC) and exhibits unprecedented PA performance. When applied to mice bearing a 6 × 3 × 3 mm^3^ tumor buried 6 mm beneath the skin, the probe 800RS-PMPC selectively accumulates in the tumor and emits PA signals that are strong enough to be unambiguously distinguished from noise signals of endogenous blood/hemoglobin. The PA image thus obtained under high-threshold conditions allows 3D characterization of the tumor in terms of its size, shape, location, and boundaries.

## Introduction

Photoacoustic (PA) imaging is a growing bioimaging modality in which light-to-heat conversion mediated by light absorbers results in thermoelastic expansion of the surrounding tissues to lead to the emission of ultrasonic waves^1,2^. Compared with fluorescence, ultrasonic waves have some fascinating features, especially for analysis with respect to depth, i.e., it shows far less (2–3 orders of magnitude) scattering by body tissues to allow deeper (several centimeters) penetration and much higher (submillimeter) spatial resolution^3–6^. Since hemoglobin is an ideally PA-relevant endogenous chromophore, PA imaging is particularly suited for high-resolution 3D (three-dimensional) imaging of the vasculature of body tissues such as skin and implanted tumors^7–9^. Another approach is to use genetic reporters such as fluorescent proteins and pigments as "semi-endogenous" contrast agents^10,11^. 3D imaging of tumors formed of cells that have been lentivirally transduced to express a reporter protein is a notable recent example^12^.

In addition to endogenous and semi-endogenous chromophores, there is a wide range of exogenous or artificial contrast agents that are absorption-tunable in the near-infrared (NIR) region (700–900 nm) and modifiable, particularly with biocompatible polymers^13–18^, so that their biofunctions, such as their circulation in blood, selectivity, and efficiency, can be improved. Recent attention has also been paid to contrast agents in the so-called second NIR (1000–1700 nm) window (NIR-II) region^19,20^ and their formulation^21^ to allow higher spatial resolution, deeper penetration, and lower interference by tissue substrates with minimal autofluorescence. Under these circumstances, PA imaging of tumors in animals has been intensely studied in recent years using various types of NIR and NIR-II^21^ contrast agents (dyes^22–24^, quantum dots^25^, nanoparticles^26–28^, semi-conducting polymers^29^, and so on^30,31^) in a variety of formulations and with diverse targeting strategies^32^. Hopefully, the tumors, even when they are small and buried deep in the body, can be three-dimensionally drawn by these probes. To the best of our knowledge, however, this potentiality has not yet been adequately demonstrated, and remains a challenge. This is not surprising since the selectivity and efficiency of tumor-targeting by artificial probes are generally low and it is by no means easy to unambiguously distinguish weak signals of the probe accumulated in the tumor from those of blood hemoglobin. In addition, in view of decay of the incident light in the depth direction, there is no a priori guarantee that probe molecules distributed in the tumor three-dimensionally are equally photo-excitable.

In this work, we studied PA imaging of a 6 × 3 × 3 mm^3^ tumor buried 6 mm beneath the skin in mice. We paid particular attention to overcome the inherent hemoglobin problem by using a highly tumor-targeting polymer conjugate of the reporter dye to enhance the probe signals and by employing high-threshold conditions to completely suppress blood hemoglobin noise. We report here that the tumor can be three-dimensionally visualized in terms of its size, shape, location, and surface/boundaries by genuine probe signals that survive under these high-threshold conditions. To the best of our knowledge, this is the first example of probe-targeting, in vivo 3D PA imaging of a relatively small tumor in relatively deep tissue. The present technique represents a powerful new approach to both basic research and (pre)clinical studies of tumors.

## Results and discussion

**Figure 1:**
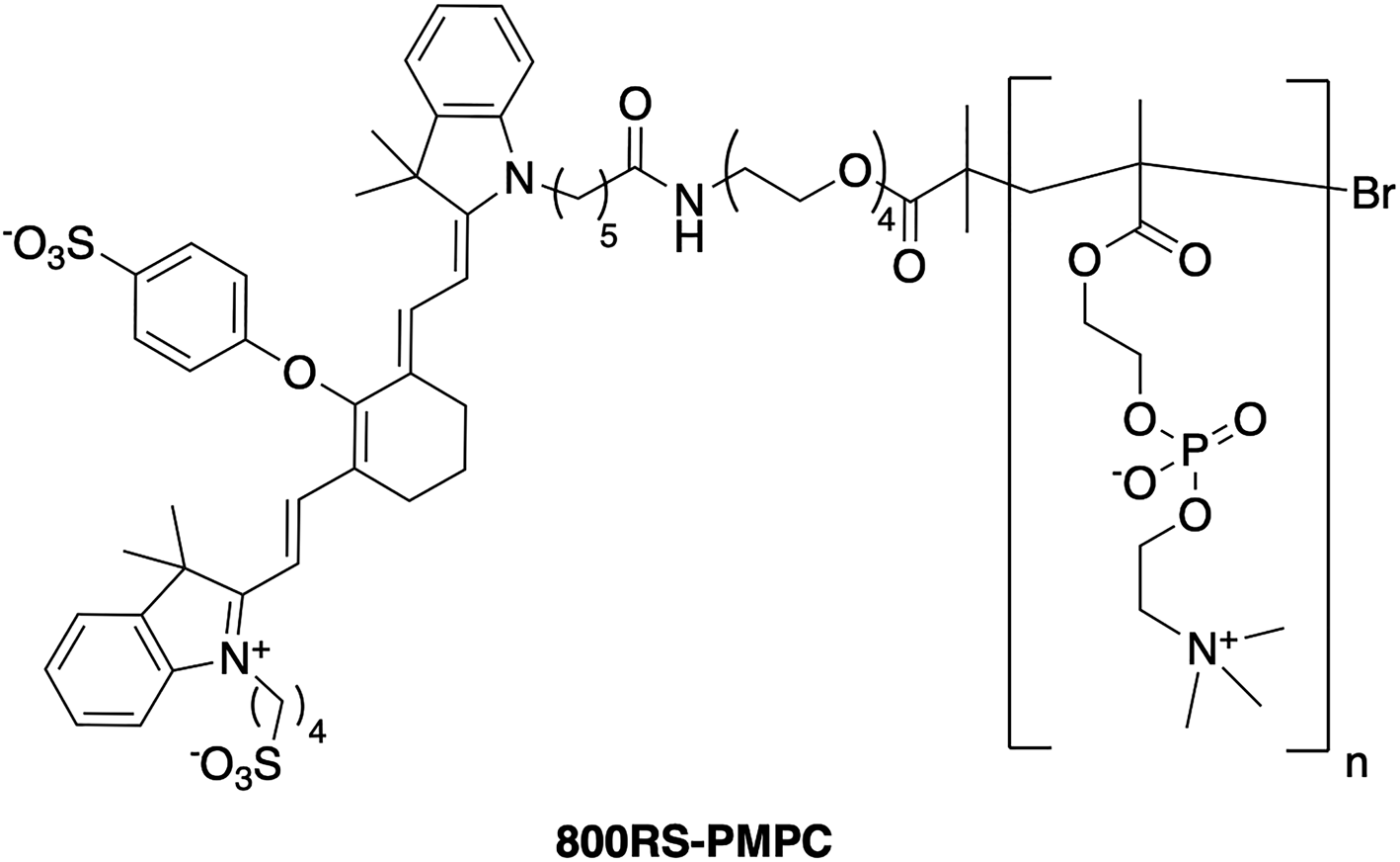
The chemical structure of 800RS-PMPC.

The PA probe used here is 800RS-PMPC (Fig. 1). PMPC, poly(2-methacryloyloxyethylphosphorylcholine)^15–17^, is highly biocompatible and selectively accumulated in tumor due to the EPR (Enhanced Permeability and Retention) effect^18^, and 800RS is a hydrophilic near-infrared (NIR) cyanine dye. 800RS was used here after we surveyed the performance of various NIR cyanine dye conjugates including its indocyanine green (ICG) counterpart. In general, ICG-conjugated polymer probes including ICG-PMPC, also prepared by our group, bound nonspecifically to bovine serum albumin (BSA), which resulted in unavoidable accumulation of ICG-conjugated polymer probes in the liver^13^. The conjugate 800RS-PMPC with a molecular weight of 56–71 kDa was obtained in a yield of ~ 50% via succinimidyl coupling of the respective precursors (see Supplementary Information). Notable instability or cytotoxicity was not observed for 800RS-PMPC during cold storage for months or years, in its absorption spectra (*λ*_max_ = 771 nm vs, *λ*_max_ = 770 nm for 800RS, Supplementary Fig. S5) upon repeated scans, or by MTT assay (see Supplementary Information and Supplementary Fig. S7) with a cell viability of ~ 100% at concentrations (≤ 100 µM) relevant to in vivo studies.

**Figure 2:**
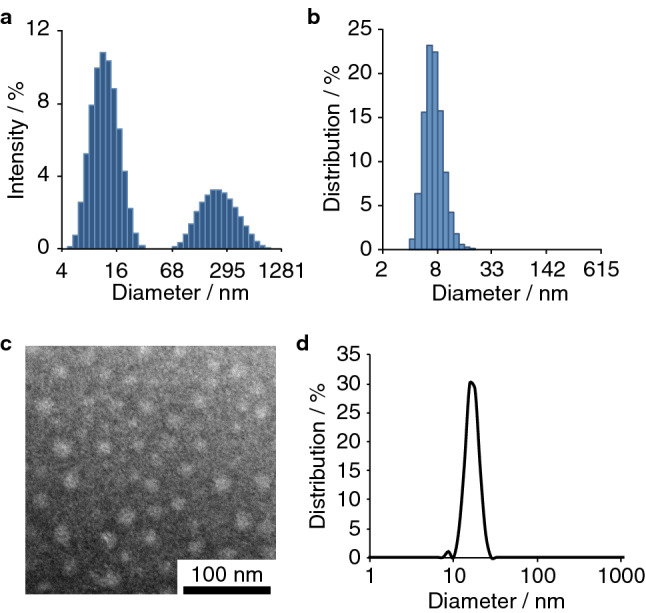
Intensity-weighted (**a**) and volume-weighted (**b**) DLS (Dynamic Light Scattering) size distribution profiles and TEM (Transmission Electron Microscopic) images (**c**) and size distribution histogram (**d**) for 800RS-PMPC. DLS samples of 800RS-PMPC (1 mg/mL H_2_O) were filtered through a 0.8 µm filter just prior to measurements. TEM samples of 800RS-PMPC (2 mg/mL H_2_O) were negatively stained with phosphotungstic acid (2.8 wt%, pH 7.0).

DLS (Dynamic Light Scattering) indicated that 800RS-PMPC has a slightly negative surface (*ζ*) potential of − 10.2 ± 2.0 mV and forms compact nanoparticles with a hydrodynamic diameter (intensity-weighted) of *d*_DLS_ = 14.3 ± 0.4 nm (Fig. 2a) in a similar manner as the unmodified parent polymer, i.e., NH_2_-PMPC (10.9 ± 0.4 nm). Bigger particles in the 200–300 nm region in Fig. 2a, i.e., in the intensity-weighted formulation (proportional to the sixth power of the diameter) are of only limited significance, since they vanish in the volume-weighted formulation (proportional to the third power of the diameter) (Fig. 2b). The TEM (Transmission Electron Microscopy) image of the conjugate (Fig. 2c) and the size distribution histogram (Fig. 2d) with a mean diameter of 15.8 ± 2.8 nm agree with the DLS size of 14.3 nm, in a similar manner as other PMPC-containing nanoparticles^33,34^. 800RS-PMPC has been shown to be highly biocompatible by QCM (Quartz Crystal Microbalance) measurements using a BSA-immobilized sensor chip, which show that 800RS-PMPC has practically no affinity for BSA (see Supplementary Fig. S8); bovine serum albumin is the most abundant serum protein. On the other hand, ICG-PMPC formed giant aggregates, bound nonspecifically to BSA, and was less selective in tumor-targeting, possibly as a consequence of the relatively hydrophobic nature of the ICG moiety as compared with its hydrophilized analogue 800RS (see Supplementary Information for details). After confirming that the characteristics of the parent polymer PMPC that make it a good EPR tumor-targeter, i.e., compact size^35–37^ and biocompatibility^38–40^, are retained in the conjugate 800RS-PMPC (but not in the conjugate ICG-PMPC), we moved on to PA imaging of tumors in mice.

**Figure 3:**
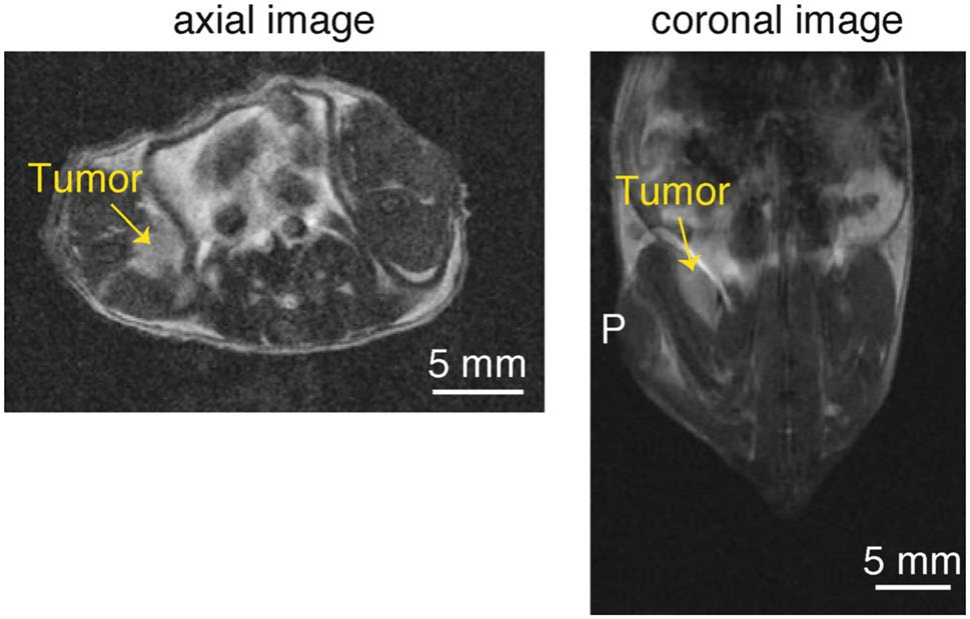
MR (Magnetic Resonance) images (axial and coronal) at 7 T for a tumor-bearing mouse 5 days after inoculation of colon 26. The tumor is marked with a yellow arrow.

**Figure 4:**
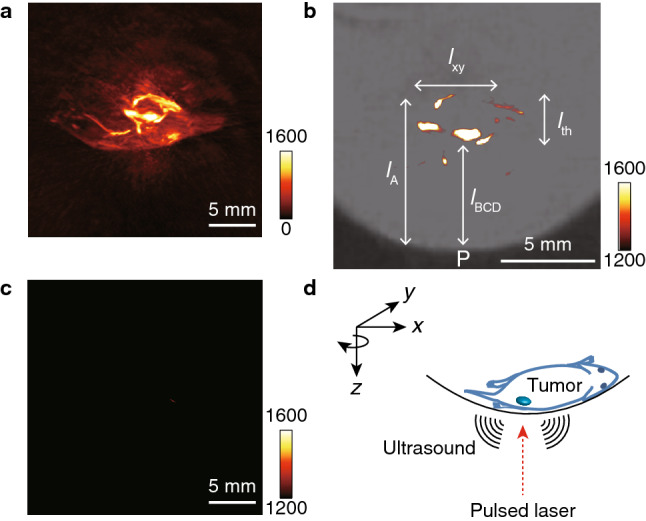
PA (Photoacoustic) images for a tumor-bearing and 800RS-PMPC-administered (2.0 µmol/kg = 40 nmol/20-g mouse) mouse under ambient high-sensitivity conditions with a sensitivity scale of 0–1600 (**a**), for a tumor-bearing and 800RS-PMPC-administered mouse as in (**a**) but under hemoglobin-suppressing, low-sensitivity, or high-threshold conditions with a sensitivity scale of 1200–1600 (**b**), and for a tumor-bearing mouse without administration of 800RS-PMPC under hemoglobin-suppressing, low-sensitivity, or high-threshold conditions (**c**) as in (**b**). The PA image in (**b**) is merged with a bright-field image. (**d**) shows a schematic representation of coordinate axes illustrating the arrangement of the object (tumor-bearing mouse) and the direction of the incident laser pulse.

**Figure 5:**
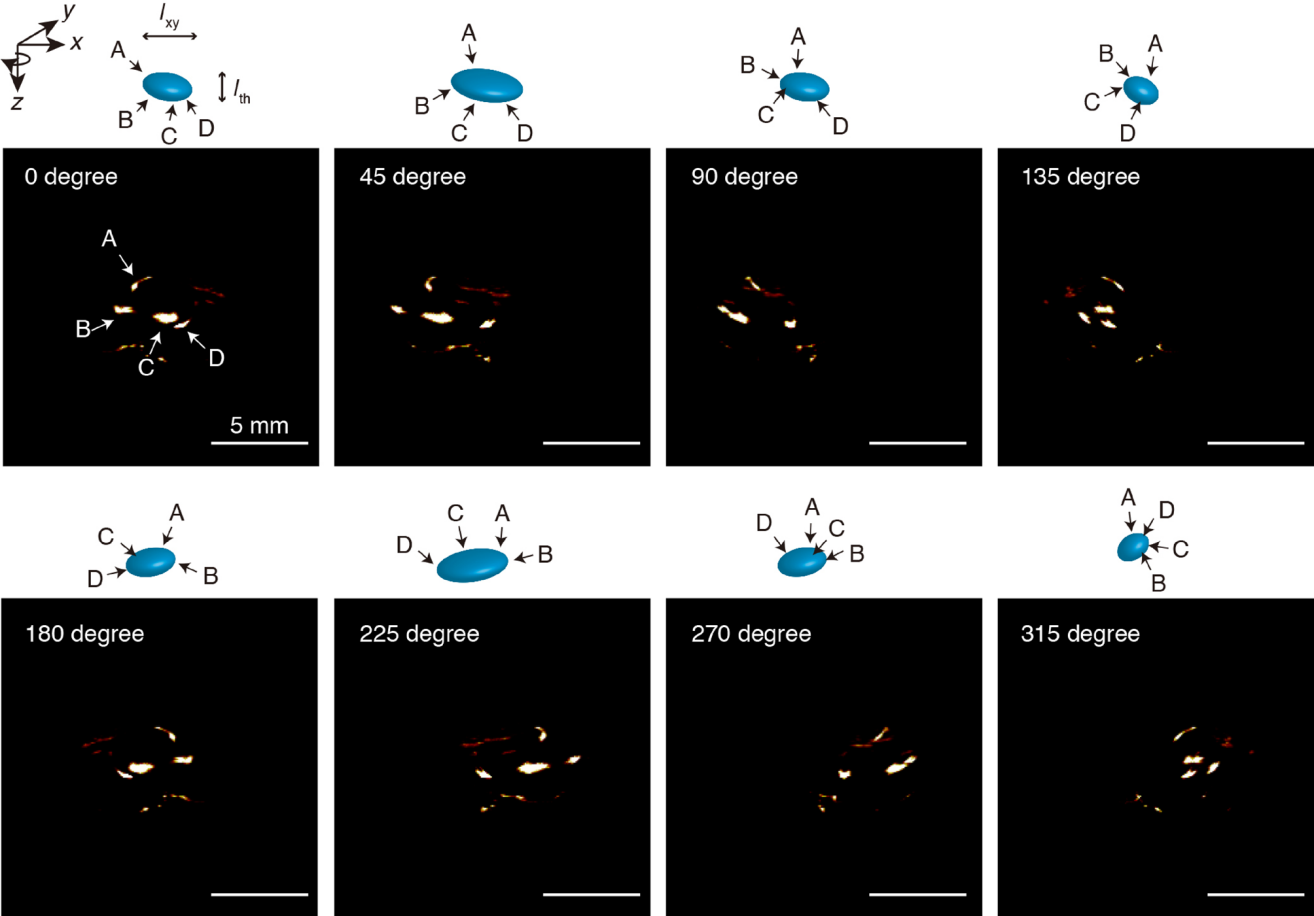
Projections on the *xz* plane (referring to the coordinates shown in Fig. 4d) of the 3D PA image of a tumor-bearing and 800RS-PMPC-administered mouse at various angles with respect to rotation around *z*, i.e., incident laser axis. The drawings illustrate how four bright spots (A, B, C, and D) within an ellipse move upon rotation. Scale bars: 5 mm.

Small tumors were injected into mice by the careful intramuscular inoculation of colon 26 cells several millimeters beneath the skin of the right leg. The growth of the tumor was monitored by 7 T MRI. After 5 days, the tumor, shown by a yellow arrow in the indistinct MRI image (Fig. 3), was found to be located ~ 6 mm beneath the skin, i.e., from the inoculation point P, and to have an ellipsoidal shape with major and minor axes of ~ 6 mm and ~ 3 mm, respectively, as shown by the coronal image, i.e., top view of the tumor-containing coronal slice (Fig. 3). The tumor could also be readily visualized by PA imaging when tumor-bearing mice were injected intravenously with 800RS-PMPC (2.0 µmol/kg = 40 nmol/20-g mouse); we did not observe acute toxicity in any of the animals. In PA imaging, the mouse was placed in the PA apparatus with the tumor site at the bottom and irradiated with a pulsed laser at 790 nm (see “Methods” for details) from below at 48 h after the administration of 800RS-PMPC. PA signals generated were collected by a 3D detector to produce 3D images. An actual PA image taken at an arbitrary high sensitivity with a scale of 0–1600 is shown in Fig. 4a. It is composed of several bright areas in addition to a less intense background which is due to blood vessels containing PA-active hemoglobin, as independently confirmed. Blood (hemoglobin) signals may be conveniently removed by subtraction of the image in the absence of the probe from that in its presence. This subtraction method is often used in the literature^30,31^. However, care should be taken when interpreting less-intense signals that survive subtraction because they can still be noise. An alternative and more persuasive/convincing approach, if possible, would be to consider only doubtless probe signals that are more intense than even the most intense endogenous signals. Under these hemoglobin-suppressing, low-sensitivity, or high-threshold conditions with a scale of 1200–1600 (see “Methods” for details), a tumor-bearing mouse treated with 800RS-PMPC exhibited a clear PA image (Figs. 4b and 5 with (4b) or without (5) a merged bright-field image) composed of at least four bright spots/areas (A, B, C, and D in the top-left panel of Fig. 5), in marked contrast to the no-signal image for a tumor-bearing but 800RS-PMPC-nontreated reference mouse under otherwise identical conditions (Fig. 4c). Splitting of bright or hot spots was observed repeatedly (n = 3) for 800RS-PMPC-administered mice (see Supplementary Fig. S9b as another example), but was not clear when ICG-PMPC as a control was used in place of 800RS-PMPC (Supplementary Fig. S9a).

With reference to the horizontal (*x*), longitudinal (*y*), and vertical (*z*) coordinates illustrated in Fig. 4d, the PA image in Fig. 4b is actually the projection of the 3D image on the *xz* plane and the vertical distance (along the laser path) from point P (the inoculation point on the skin) represents depth. Thus, referring to Fig. 4b, the shallow three bright spots (B, C, and D in the top-left panel of Fig. 5) are embedded inside the mouse body with a depth (from point P) of *l*_BCD_ =  ~ 5 mm and the fourth spot (A) is buried deeper (*l*_A_ =  ~ 8 mm) and is less bright. The four bright spots as a whole are accommodated within a volume centered at ~ 6 mm deep from the skin and with a thickness of *l*_th_ =  ~ 3 mm and an apparent *xy* or horizontal/longitudinal width of *l*_*xy*_ =  ~ 4 mm. Assuming that the PA signals come from the probe, i.e., 800RS-PMPC, its distribution in the *xy* (horizontal/longitudinal) plane becomes clearer if we rotate the viewpoint around the *z* (laser path) axis, referring to Fig. 4d. The PA images at tilt angles of 0°, 45°, 90°, 135°, 180°, 225°, 270°, and 315° are shown in Fig. 5 (see Supplementary Information for a movie). During rotation, the thickness (*l*_th_) of the volume that encompasses the four bright spots remains constant at ~ 3 mm, as expected. On the other hand, the apparent *xy* (horizontal/longitudinal) width (*l*_*xy*_) of the four bright spots overall changes with changes in the tilt angles and is shortest (3 mm) at 135° and 315°, longest (6 mm) at 45° and 225°, and intermediate at 90° and 180° (Fig. 5). These results leave little doubt that the four bright spots are contained within the same volume (see the drawings in Fig. 5), which has an ellipsoidal shape with a major axis of 6 mm, a minor axis of 3 mm, and a thickness of 3 mm and is centered at ~ 6 mm from the skin. The location, shape, and size of this ellipse are in excellent agreement with those of the MRI-visualized tumor (~ 6 mm × ~ 3 mm, centered at ~ 6 mm from the skin, referring to Fig. 3). We are thus led to conclude that the PA signals obtained under the hemoglobin-suppressing, low-sensitivity, or high-threshold conditions come from the probe 800RS-PMPC distributed not only on the front surface (B, C, and D) but also on the rear surface (A) of the tumor, and thereby they define the surface or boundary and, as a consequence, allow us to draw a 3D image of the tumor *to the same extent as MRI visualization of the tumor*. While the non-uniform distribution of the intense PA signals of the probe on the tumor surface is not readily explainable, it is by no means surprising since there is no reason to expect that new blood vessels from which the probe leaks into the tumor by the EPR effect should be uniformly formed at the tumor site. Nevertheless, the present work raises the question of the apparent localization of the probe on the surface and not inside of the tumor. To answer this question, we need to know more not only about the intra-tumor trafficking of this type of polymeric probe, but also about the dependency of PA intensity under the present high-threshold conditions on the local environment, particularly microviscosity or microfluidity.

## Conclusions

In summary, this study demonstrated that high-quality, MRI-consistent, and *much clearer* 3D anatomical images of a 6 × 3 × 3 mm^3^ tumor buried 6 mm beneath the skin were obtained by probe-targeting in vivo PA imaging. This method may provide a powerful new approach to 3D monitoring of the morphological changes of tumors associated with, for example, their development/growth and response to therapy. Clinically, a critical issue in tumor surgery is tumor thickness. The present tool may illustrate a PA approach to the operative or intraoperative monitoring of a tumor to be removed.

A methodological advantage or innovation of this work lies in the setting of a high threshold to completely suppress the hemoglobin noise signals. Independent PA imaging of deoxyhemoglobin (Hb) and oxyhemoglobin (HbO_2_) in a blood vessel to measure the oxygen saturation of hemoglobin (sO_2_) has been established within the last decade^1,2^. However, PA signals of hemoglobin act as endogenous noise signals to obtain 3D PA images of the tumor surrounded by the probe. Accordingly, the PA signals of the probe accumulated in the tumor site must be strong enough to survive under hemoglobin-suppressing, high-threshold conditions, where the high tumor-targeting ability of the PMPC backbone of the probe, 800RS-PMPC, must play an important role. Indeed, ex vivo studies with fluorescence quantification showed that the 800RS-PMPC probe selectively accumulated in the tumor with an efficiency of 11.6% ID/g (ID = injected dose), as compared with 13% ID/g for the doubly ^13^C/^15^N-labeled and self-traceable PMPC^18^. However, this is not the whole story. It is also important that the incident laser light is not consumed at the front surface of the tumor, but rather passes through it to reach the rear surface to excite the probe there to give information about the rear surface. In this context, we have to shed more light on the photophysics of the probe and the site-dependency of the PA intensity thereof. While various contributing factors remain to be systematically analyzed, the present work opens the door to the sophisticated use of high-contrast in vivo PA imaging for tumor tomography and illustrates some key properties the probe should have for that purpose. Further work along these lines is now underway.

## Methods

### General

NMR spectra were obtained on a Bruker Avance-500 (500 MHz) spectrometer with solvent peaks as references. GPC analyses were conducted at 40 °C on a JASCO LC-Net II/ADC system equipped with a Shodex SB-803HQ column. An aqueous solution of NaNO_3_ (0.1 M) with NaN_3_ (0.2 wt%) was used as an eluent at a flow rate of 1.0 mL/min. Absorption spectra were recorded using a HITACHI U-3010 UV/Vis spectrophotometer equipped with a Peltier device (EHC-573T). Dynamic Light Scattering (DLS) and *ζ* potential measurements were performed for aqueous solutions (1 mg/mL) of PMPC samples on a Malvern Zetasizer Nano-ZS analyzer (equipped with a ZEN 9062 filter) with a 4 mW He–Ne laser at 633 nm. Transmission Electron Microscopy (TEM) images were obtained with a JEOL JEM-1400 microscope at an acceleration voltage of 100 kV. A solution (2 mg/mL) of PMPC samples in water was dispersed onto hydrophilic copper grids, and excess solution was removed with filter paper. The grids were immersed in phosphotungstic acid (2.8 wt%, pH 7.0) as a negative staining agent, and then subjected to TEM measurements. The TEM images were analyzed using ImageJ software to give size distribution histograms. Photoacoustic (PA) imaging was conducted with an Endra Nexus 128 preclinical photoacoustic computed tomography scanner, and the PA images were reconstructed and analyzed using OsiriX software (Newton Graphics) according to the manufacturer’s instructions. ^1^H MR images of mice were obtained using a Bruker 7T MR system (Biospec 70/20 USR) as described elsewhere^18^.

### Preparation and properties of 800RS-PMPC and ICG-PMPC

800RS-PMPC was obtained as shown in Supplementary Scheme S1. The number average molecular weights (*M*_n_) of 800RS-PMPC conjugates were estimated from the ratios of NMR integration for the methylene protons of the repeating MPC units in the PMPC polymer chain to that for the aromatic protons in the dye moiety at the terminus of the conjugates. Polydispersity indices (*M*_w_/*M*_n_) were evaluated from GPC profiles calibrated with poly(ethylene glycol) standards.

### Fmoc-PMPC

Fmoc-PMPC was obtained by ATRP (Atom Transfer Radical Polymerization) of 2-methacryloyloxyethylphosphorylcholine (MPC) using Fmoc-protected bromoisobutylate derivative (Fmoc-initiator)^41^ as an initiator under manipulated control of the initiator/monomer ratios. Thus, in a typical preparation, CuBr (2.9 mg, 0.020 mmol) and 2,2′-bipyridine (6.16 mg, 0.039 mmol) as catalysts were added to a solution of MPC (511.6 mg, 1.73 mmol) and Fmoc-protected initiator (5.8 mg, 0.010 mmol) in degassed methanol (1.0 mL) in a nitrogen glove box. The mixture was stirred at room temperature for 4 days. When the reaction was complete, the solution was passed through a thin silica gel layer with methanol as an eluent. The eluate was concentrated, and anhydrous methanol and THF were added to the residue. The polymeric product that precipitated was collected, washed with anhydrous THF, dried under reduced pressure, purified by ultrafiltration using a centrifugal filter with a cut-off molecular weight of 10 kDa, and lyophilized to give Fmoc-PMPC as a white powder in a yield of 63%; ^1^H NMR (500 MHz, CD_3_OD) *δ*: 7.83 (*d*, 2H, *J* = 7.3 Hz), 7.67 (*d*, 2H, *J* = 6.6 Hz), 7.37–7.45 (*m*, 2H), 7.30–7.37 (*m*, 2H), 4.28–4.40 (br *m*, ~ 400H), 4.14–4.28 (br *m*, ~ 280H), 3.98–4.14 (br *m*, ~ 350H), 3.67–3.77 (br *m*, ~ 390H), 3.22–3.33 (br *m*), 1.40–2.30 (br *m*, ~ 360H), 0.70–1.26 (br *m*, ~ 580H).

### NH_2_-PMPC

A solution of Fmoc-PMPC (149.8 mg, 2.7 µmol) and DBU (20.5 µL, 0.14 mmol) in methanol (1.0 mL) was stirred for 3 h at room temperature. The progress of the reaction was checked by ninhydrin colorimetry. The solution was concentrated, and the residue was reprecipitated with anhydrous methanol and THF. The precipitate was collected and dried under reduced pressure to give deprotected NH_2_-PMPC as a white powder in a quantitative yield; ^1^H NMR (500 MHz, D_2_O) *δ*: 4.22–4.31 (br *m*, ~ 420H), 4.10–4.22 (br *m*, ~ 350H), 3.95–4.10 (br *m*, ~ 380H), 3.61–3.68 (br *m*, ~ 390H), 3.16–3.27 (br *m*, ~ 1850H), 1.50–2.15 (br *m*, ~ 400H), 0.63–1.20 (br *m*, ~ 600H). The absence of the characteristic signals for the 9-fluorenyl group of Fmoc at 7.83, 7.67, 7.37–7.45, and 7.30–7.37 in the NH_2_-PMPC product confirmed complete deprotection of the precursor Fmoc-PMPC.

### 800RS-PMPC

800RS-PMPC was obtained by the coupling of NH_2_-PMPC and the corresponding *N*-hydroxysuccinimidyl (NHS) ester of 800RS, i.e., IRDye 800RS NHS ester (800RS-NHS; Li-Cor). A typical procedure is as follows. NH_2_-PMPC (*M*_n_ = 54,000) (0.57 µmol) and 800RS-NHS (1.03 µmol) were dissolved in anhydrous methanol and the mixture was stirred for 44–48 h at room temperature in the dark. The solution was ultrafiltrated using a centrifugal filter unit with a cut-off molecular weight of 10 kDa, and further purified with a Sephadex G25 column (PD-10, GE Healthcare) to give 800RS-PMPC (*M*_n_ = 56,000, *M*_w_/*M*_n_ = 1.66) in a yield of 54%; ^1^H NMR (CD_3_OD, 500 MHz) *δ*: 7.88 (*d*, 1H, *J* = 13.7 Hz), 7.82 (*d*, 1H, *J* = 14.2 Hz), 7.73–7.78 (*m*, 2H), 7.20–7.33 (*m*, 5H), 7.04–7.17 (*m*, 5H), 6.16 (*d*, 1H, *J* = 14.1 Hz), 6.04 (*d*, 1H, *J* = 14.8 Hz), 4.18–4.29 (br *m*, ~ 360H), 4.04–4.18 (br *m*, ~ 250H), 3.88–4.04 (br *m*, ~ 330H), 3.60–3.70 (br *m*, ~ 380H), 3.22–3.33 (br *m*), 1.30–2.30 (br *m*, ~ 360H), 0.60–1.16 (br *m*, ~ 560H); absorption and fluorescence maxima (2 µM in H_2_O) 771 nm and 788 nm (excitation at 771 nm), respectively.

### ICG-PMPC

The indocyanine green conjugate of PMPC (ICG-PMPC) was obtained in a yield of 70% in a similar manner as 800RS-PMPC, i.e., by coupling of NH_2_-PMPC and the corresponding *N*-hydroxysulfosuccinimidyl ester of ICG (ICG-Sulfo-OSu; Dojindo). (*M*_n_ = 56,000, *M*_w_/*M*_n_ = 1.28); ^1^H NMR (CD_3_OD, 500 MHz) *δ*: 8.19–8.26 (*m*, 2H), 7.95–8.08 (*m*, 6H), 7.44–7.67 (*m*, 7H), 6.55–6.67 (*m*, 2H), 6.27–6.46 (*m*, 2H), 4.28–4.40 (br *m*, ~ 380H), 4.14–4.28 (br *m*, ~ 280H), 3.97–4.14 (br *m*, ~ 340H), 3.67–3.77 (br *m*, ~ 380H), 3.22–3.33 (br *m*), 1.40–2.30 (br *m*, ~ 380H), 0.70–1.26 (br *m*, ~ 550H); absorption and fluorescence maxima (2 µM in H_2_O) 786 nm and 805 nm (excitation at 786 nm), respectively.

The ^1^H NMR spectra for Fmoc-PMPC, NH_2_-PMPC, 800RS-PMPC, and ICG-PMPC, and the absorption spectra for 800RS-PMPC and 800RS are shown in Supplementary Figs. S1–S5.

### Cell culture

The mouse colon carcinoma cell line (Colon 26) and the mouse fibroblasts (L929) were supplied by Prof. Yasuhiko Tabata (Kyoto University, Japan) and maintained in 10% FBS-RPMI1640 medium and 10% FBS-D-MEM medium, respectively. Cells were incubated in a well-humidified incubator with 5% CO_2_ and 95% air at 37 °C.

### MTT assay of the cytotoxicity of 800RS-PMPC

The cytotoxicity of 800RS-PMPC was evaluated (n = 5) by MTT assay at 0.01–100 µM. This concentration range covers the blood level of 800RS-PMPC in in vivo studies, where the dose of 800RS-PMPC (40 nmol/20-g mouse) corresponds to a blood level of [800RS-PMPC] = 20 µM (assuming that the volume of the blood of a mouse is ~ 2 mL). Cells (L929 mouse fibroblasts) were seeded at a density of 1.0 × 10^4^ cells/cm^2^ in a 96-well plate and cultivated. After 24 h, the medium was replaced by a fresh medium containing 800RS-PMPC, and the cells were incubated for 48 h at 37 °C. After removal of the medium, the cells were washed with PBS three times and subjected to MTT assay for viable cells.

### Ethics of animal experiments

All animal experiments using BALB/cSlc-nu/nu mice (6-week-old, female, Japan SLC Inc.) were approved by the animal experiment committee of the Faculty of Engineering and Radioisotope Research Center at Kyoto University, and performed according to the Institutional Guidelines of Kyoto University. At the end of the experiments, the mice were sacrificed by an overdose of sodium pentobarbital. The tissues were rapidly removed, frozen in liquid nitrogen and stored at − 80 °C.

### QCM analysis of the interaction of 800RS-PMPC and ICG-PMPC with BSA

A quartz crystal microbalance (QCM) analysis was performed on an AFFNIX-Q QCM system. A solution of 800RS-PMPC or ICG-PMPC (500 molar equivalents of immobilized BSA) in PBS buffer was slowly added to the quartz crystal sensor immobilizing BSA (159 ~ 338 fmol). Changes in frequency (*ΔF*), normalized to the initial changes (*ΔF*_BSA_) upon immobilization of BSA, were recorded on the QCM system until adsorption was saturated (see Supplementary Fig. S8).

### Photoacoustic imaging of tumor-bearing mice

Colon 26 cells (2 × 10^5^ cells) suspended in a 50% Geltrex-PBS solution (3 µL) were injected intramuscularly into the right leg of female BALB/cSlc-nu/nu mice. MR images of the mice 5 days after inoculation showed that the tumor had developed in deep tissue and were of an appropriate size. Next, the mice were administered 800RS-PMPC (40 nmol in 200 µL of saline) intravenously via the tail vein, set in the PA apparatus, and irradiated from below with a laser pulse (790 nm) at 48 h after the administration of 800RS-PMPC. We selected the excitation wavelength of 790 nm, which was shifted toward a longer wavelength by ~ 20 nm from the absorption maximum (771 nm) of 800RS-PMPC, to reduce the contribution of hemoglobin (deoxyhemoglobin) which still shows significant absorption in this region (770–800 nm) with decreasing absorbance at longer wavelengths. PA images were acquired using the following acquisition parameters: number of angles = 120, number of pulses per angle = 20, laser intensity = 7.2–7.6 mJ, and the volume of interest = 25 × 25 × 25 mm^3^. PA images (2D/3D) and 3D movie data (Supplementary Movie) were reconstructed using OsiriX software and by manipulating the window level (WL), corresponding to brightness, and window width (WW), corresponding to contrast. Setting these at WL = 800 and WW = 1600 to give a sensitivity scale of 0–1600 represents high-sensitivity conditions (Fig. 4a). Setting these at WL = 1400 and WW = 400 to give a scale of 1200–1600 represents low-sensitivity or high-threshold conditions (Figs. 4b,c, 5, and Supplementary Movie). The body temperature of the mice was maintained by using a water-heating system at 38 °C. During the measurement, the mouse was under anesthesia with 1–2% isoflurane in air.

### Ex vivo fluorescence imaging of tumor-bearing mice treated with 800RS-PMPC and ICG-PMPC

Female BALB/cSlc-nu/nu mice at 6 weeks of age were inoculated subcutaneously with a 50% Geltrex-PBS solution (50 µL) of 1 × 10^6^ cells of the cell line colon 26 at the right shoulder 7–8 days prior to the experiments. A tumor-bearing mouse weighing ~ 20 g was injected intravenously, under anesthesia with 1–2% isoflurane in air, with a PBS solution (200 µL) of 800RS-PMPC or ICG-PMPC (1.0 µmol/kg = 20 nmol/mouse) via the tail vein. The mouse was sacrificed by an overdose of sodium pentobarbital 48 h after administration of the probe. Tissues (tumor, liver, kidney, heart, and spleen) were collected and weighed. The fluorescence images (see Supplementary Fig. S10) were obtained using an IVIS Spectrum (Xenogen) in vivo imaging system, and the images were analyzed using Living Image 4.3.1 software (Xenogen) according to the manufacturer’s instructions.

## Supplementary information

Supplementary Information.

Supplementary Video.
