# Supplementary material for: Photoacoustic in vivo 3D imaging of tumor using a highly tumor-targeting probe under high-threshold conditions
Source: Sci Rep. 2020 Nov 9;10:19363. doi: 10.1038/s41598-020-76281-1 (PMC7652936; doi:10.1038/s41598-020-76281-1)
Supplement: Supplementary file 1 — Supplementary Information. [file 41598_2020_76281_MOESM1_ESM.pdf]

Supplementary Information

for

Photoacoustic *In Vivo* 3D Imaging of Tumor Using a Highly Tumor-Targeting Probe under High-Threshold Conditions

Hisatsugu Yamada[1,†]*, Natsuki Matsumoto[1], Takanori Komaki[1], Hiroaki Konishi[1], Yu Kimura[1], Aoi Son[1], Hirohiko Imai[2], Tetsuya Matsuda[2], Yasuhiro Aoyama[1]*, and Teruyuki Kondo[1]*

[1]Department of Energy and Hydrocarbon Chemistry, Graduate School of Engineering, Kyoto University, Katsura, Nishikyo-ku, Kyoto 615-8510, Japan, [2]Department of Systems Science, Graduate School of Informatics, Kyoto University, Yoshida-honmachi, Sakyo-ku, Kyoto 606-8501, Japan

*e-mail: teruyuki@scl.kyoto-u.ac.jp (T.K.); aoyama.yasuhiro.78z@st.kyoto-u.ac.jp (Y.A.); yamada.hisatsugu@tokushima-u.ac.jp (H.Y.)

[†]Present addresses: Graduate School of Technology, Industrial and Social Sciences, Tokushima University.

Contents

1. **Preparation and properties of 800RS-PMPC and ICG-PMPC**
2. **MTT assay of the cytotoxicity of 800RS-PMPC**
3. **QCM analysis of the interaction of 800RS-PMPC and ICG-PMPC with BSA**
4. **PA imaging of tumor-bearing mice treated with ICG-PMPC**
5. ***Ex vivo* fluorescence imaging of tumor-bearing mice treated with 800RS-PMPC and ICG-PMPC**
6. **References**

1. Preparation and properties of 800RS-PMPC and ICG-PMPC

Scheme S1.

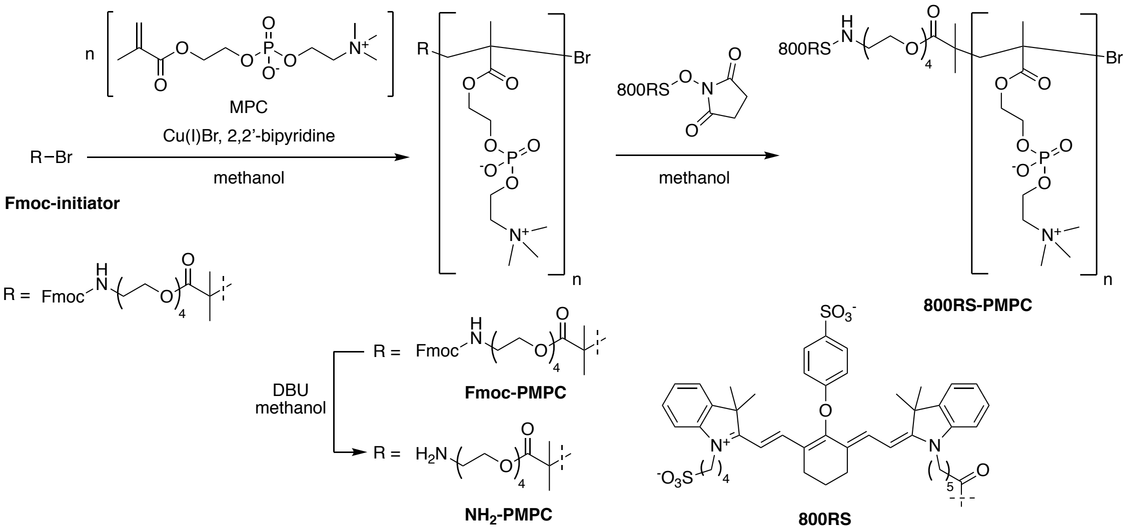

The ^{1}H NMR spectra for Fmoc-PMPC, NH_2-PMPC, 800RS-PMPC, and ICG-PMPC, and the absorption spectra for 800RS-PMPC and 800RS are shown below in Figs. S1–S5.

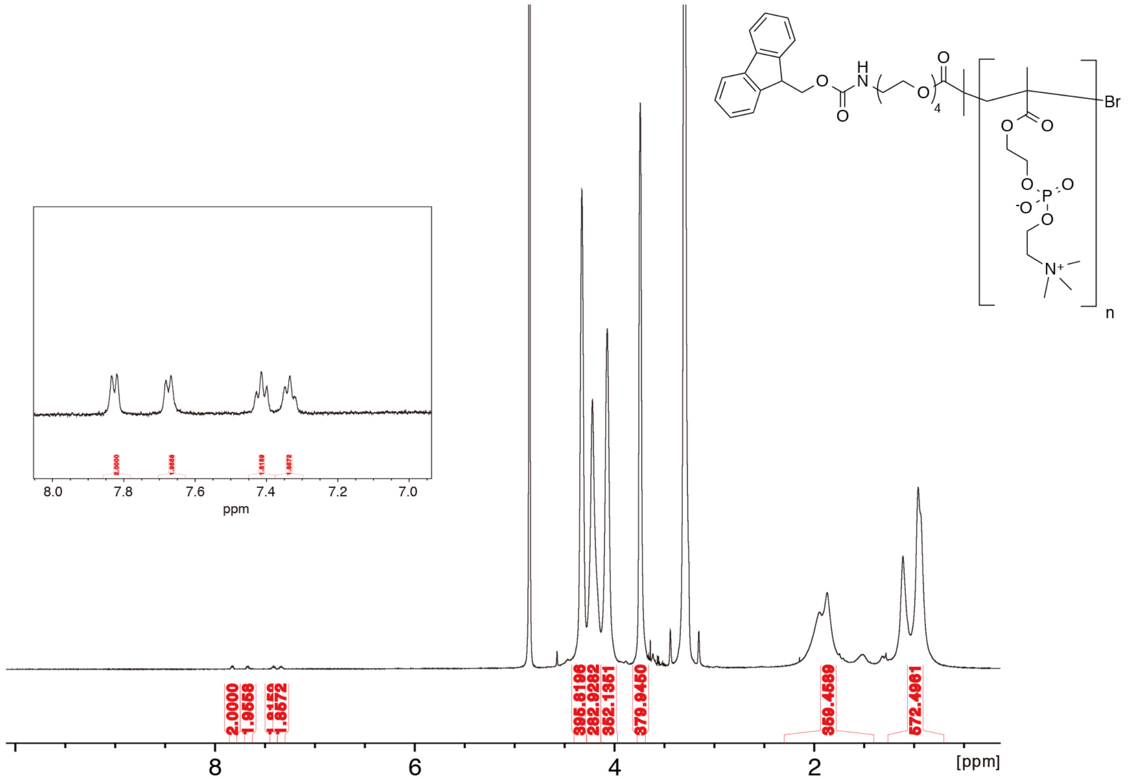

Figure S1. ^{1}H NMR spectrum of Fmoc-PMPC (M_n = 54,000) in CD_3OD (500 MHz). The inset shows the 7.0–8.0 ppm region for the Fmoc aromatic protons.

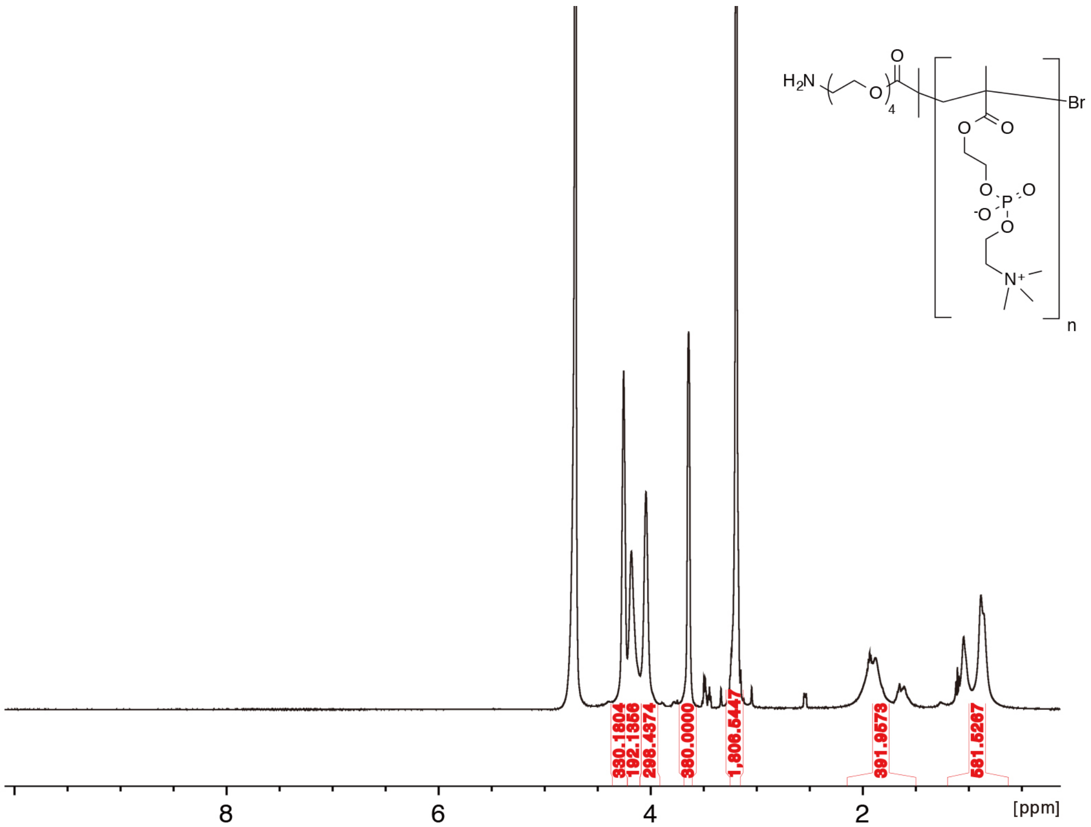

Figure S2. ^{1}H NMR spectrum of NH_2-PMPC (M_n = 54,000) in D_2O (500 MHz).

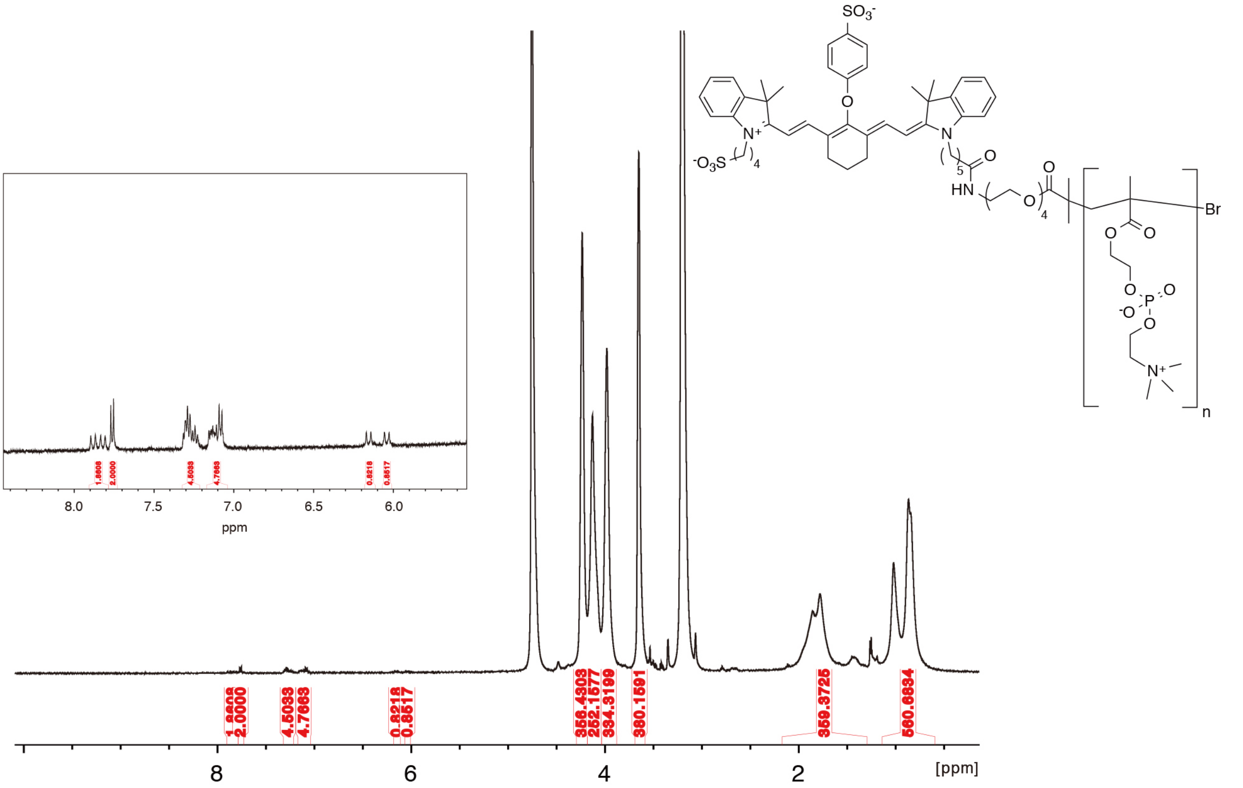

Figure S3. ^{1}H NMR spectrum of 800RS-PMPC (M_n = 56,000) in CD_3OD (500 MHz). The inset shows the 5.5–8.5 ppm region for the aromatic and olefinic protons of 800RS.

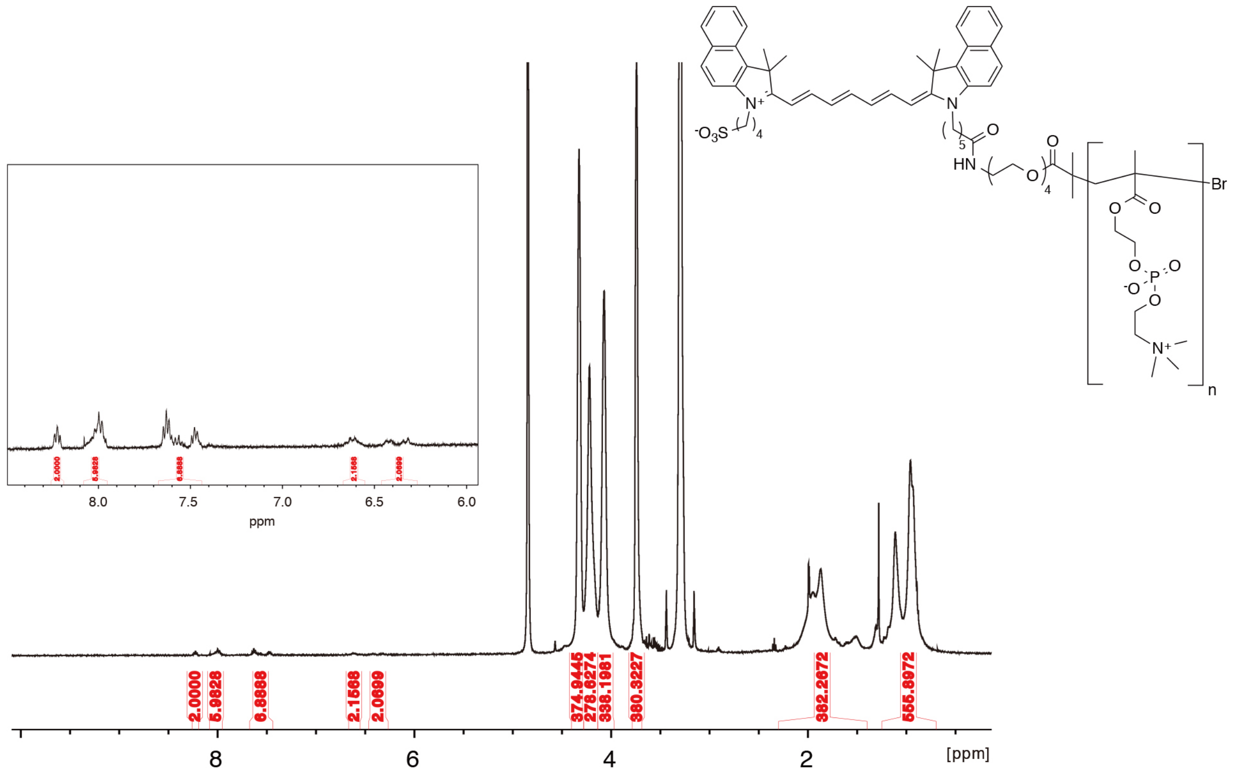

Figure S4. ^{1}H NMR spectrum of ICG-PMPC (M_n = 55,000) in CD_3OD (500 MHz). The inset shows the 6.0–8.5 ppm region for the aromatic and olefinic protons of ICG.

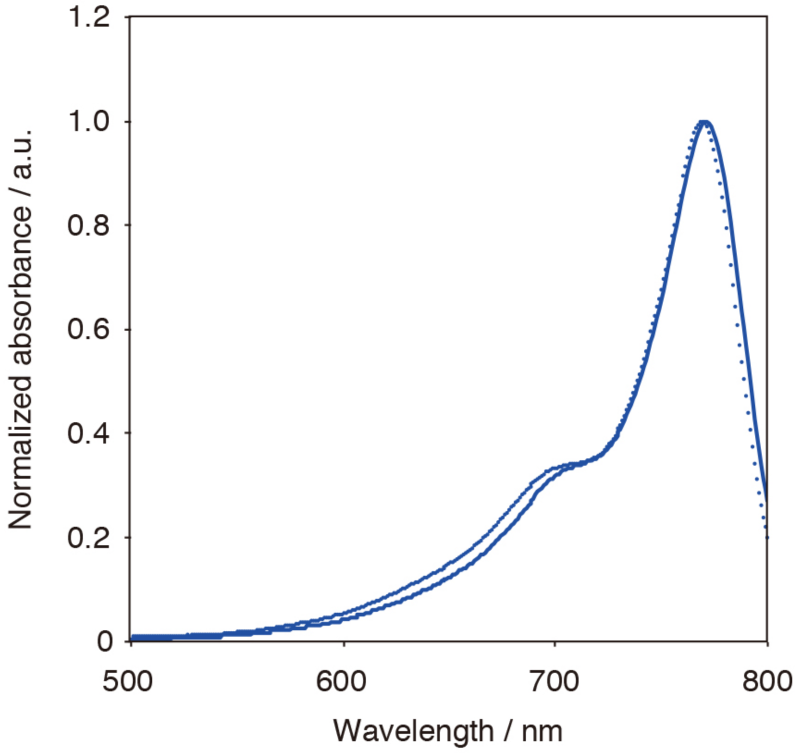

Figure S5. Absorption spectra of 800RS-PMPC (solid line) and 800RS (dotted line) in water (2 μM).

The DLS and TEM data for ICG-PMPC obtained under conditions that were otherwise identical to those for 800RS-PMPC are shown in Fig. S6 together with those for 800RS-PMPC (Fig. 1, reproduced for comparison). Thus, in marked contrast to 800RS-PMPC, ICG-PMPC forms giant aggregates (d_{DLS} = 148 ± 0.6 nm), consistent with the TEM images.

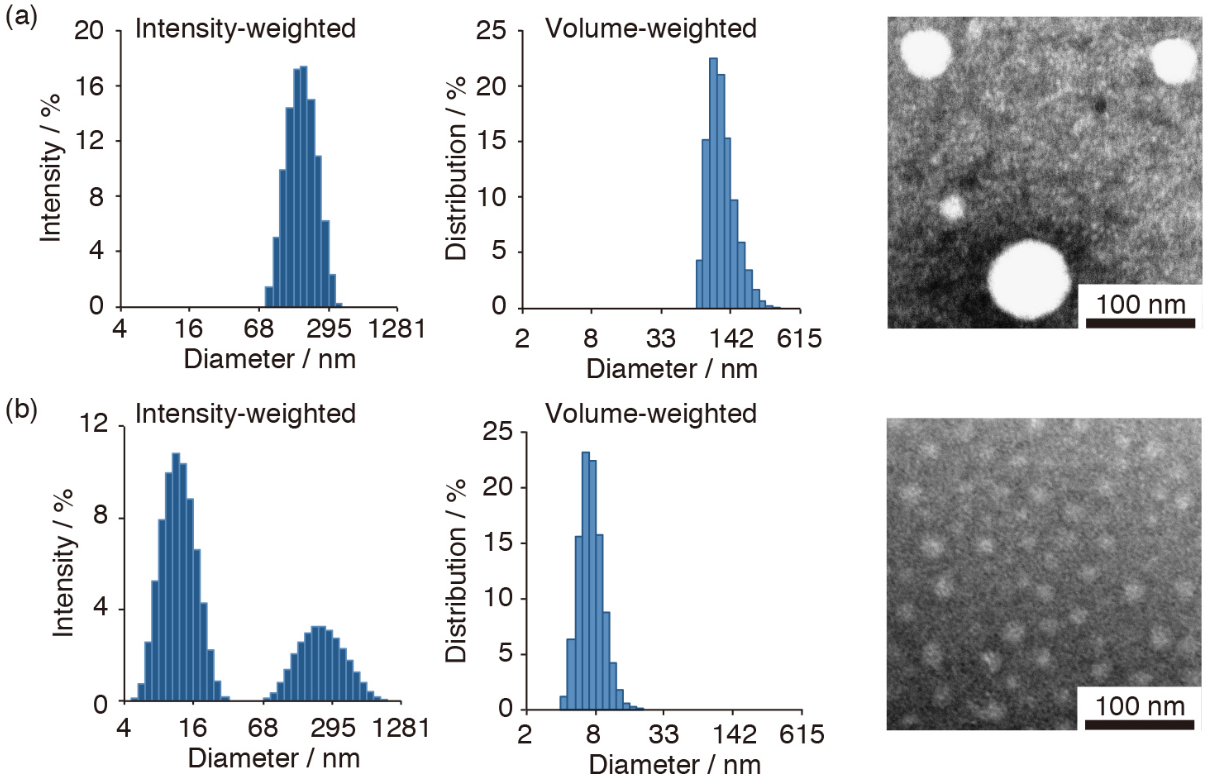

Figure S6. Intensity-weighted (left) and volume-weighted (middle) DLS size-distribution profiles and TEM images (right) for ICG-PMPC (a) and 800RS-PMPC (b, Fig. 1 reproduced). DLS samples (1 mg/mL H_2O) were filtered through a 0.8 μm filter just prior to measurements. TEM samples (2 mg/mL H_2O) were negatively stained with phosphotungstic acid (2.8 wt%, pH 7.0).

2. MTT assay of the cytotoxicity of 800RS-PMPC

The cell viabilities, shown in Fig. S7 (diamonds) are ~100% in the whole concentration range; the cytotoxicity of 800RS-PMPC if any must be very low as a consequence. In Fig. S7 are also shown the cell viabilities when NH_2-PMPC as a reference was used in place of 800RS-PMPC (squares).

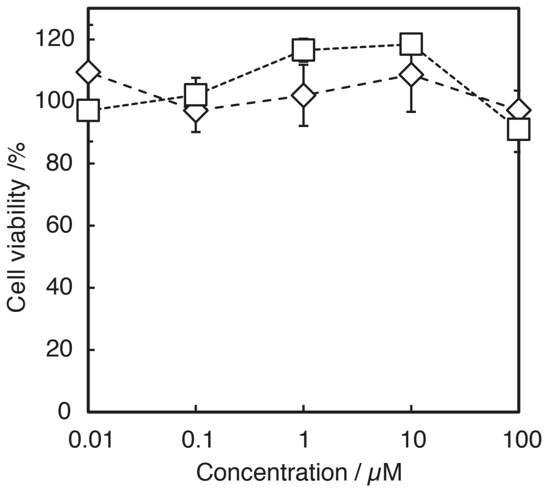

Figure S7. Cell viability assay (mean ± SD, n = 5) for 800RS-PMPC (diamond) and NH_2-PMPC (square) at 0.01–100 μM.

3. QCM analysis of the interaction of 800RS-PMPC and ICG-PMPC with BSA

Conjugate 800RS-PMPC shows no detectable affinity for BSA on the sensor. In marked contrast to 800RS-PMPC, ICG-PMPC shows a noticeable affinity for serum albumin (Fig. S8).

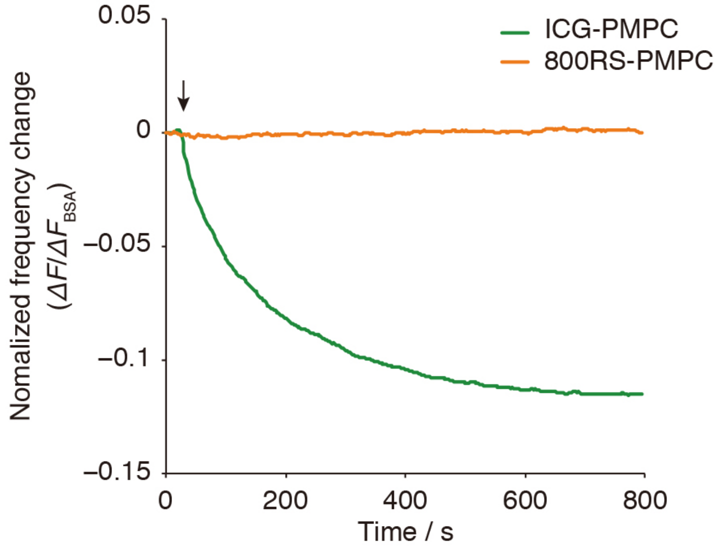

Figure S8. QCM sensorgrams for the adsorption of 800RS-PMPC and ICG-PMPC on a gold electrode immobilizing bovine serum albumin (BSA) at 25 ºC. Time-courses of normalized changes in frequency ($\Delta F/\Delta F_{BSA}$) after addition (shown by an arrow) of 800RS-PMPC (orange) or ICG-PMPC (green) (500 molar equivalents of immobilized BSA) to the electrode. ΔF_{BSA} refers to the change in frequency upon immobilization of BSA.

4. PA imaging of tumor-bearing mice treated with ICG-PMPC

A PA image for a tumor-bearing and ICG-PMPC-administered mouse under conditions that were otherwise identical to those for 800RS-PMPC is shown in Fig. S9a, which exhibits a rather continuous bright area. This is in contrast to the PA image of 800RS-PMPC-administered mouse characterized by split bright (hot) spots as shown in Figs. 4b and 5 and also in Fig. S9b as an additional example with a scale of either 1200–1600 or more clearly 1200–1400.

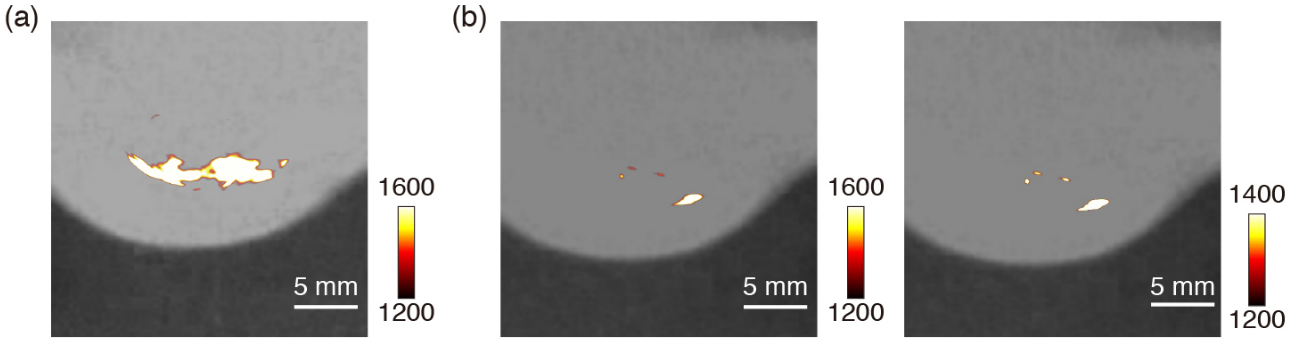

Figure S9. PA images of tumor-bearing ICG-PMPC-administered (a) or 800RS-PMPC-administered (2.0 µmol/kg = 40 nmol/20-g mouse) mice (b) under hemoglobin-suppressing, low-sensitivity, or high-threshold conditions.

5. *Ex vivo* fluorescence imaging of tumor-bearing mice treated with 800RS-PMPC and ICG-PMPC

In the case of 800RS-PMPC, strong fluorescence was detected from the tumor (Tu), in marked contrast to other tissues. When ICG-PMPC was used in place of 800RS-PMPC, strong fluorescence was detected for the liver in addition to the tumor. The tumor-to-liver fluorescence intensity ratios were 3.7 for 800RS-PMPC and 1.9 for ICG-PMPC. Quantification revealed that the probe 800RS-PMPC accumulates in the tumor with an efficiency of 4.62 nmol/g or 11.6% ID/g (ID = injected dose), as compared with 13% ID/g for the doubly $^{13}C/^{15}N$-labeled and self-traceable PMPC.[S1] Thus, 800RS-PMPC turned out to be more selective in tumor-targeting than ICG-PMPC.

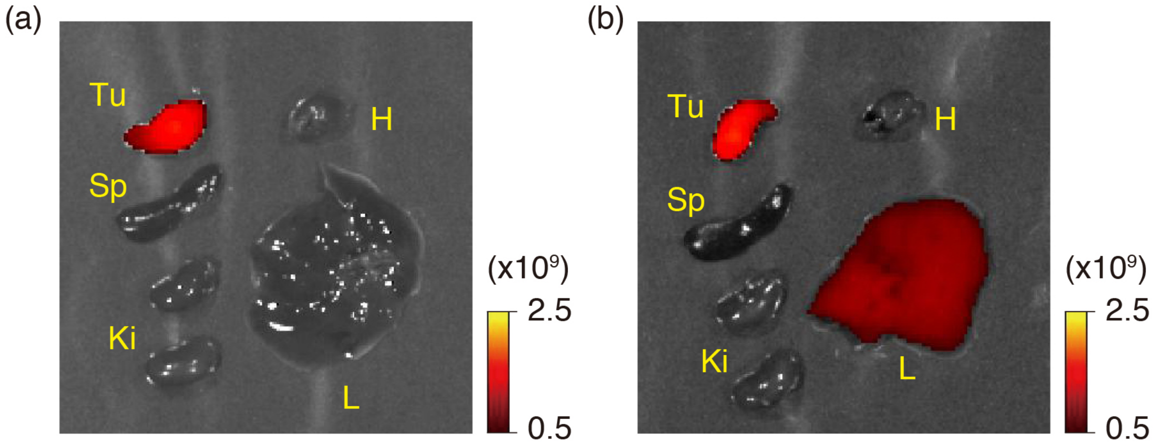

Figure S10. Merged fluorescence and bright-field images for the tumor (Tu), liver (L), kidney (Ki), spleen (Sp), and heart (H) taken from mice 48 h after administration of 800RS-PMPC (a) or ICG-PMPC (b) (1.0 μmol/kg = 20 nmol/20-g mouse). Excitation and detection wavelengths are 745 nm and 850 nm, respectively. The scale shows radiant efficiency ([photon/sec/cm^2/sr]/[μW/cm^2]).

6. References

[S1] Yamada, H. *et al.* Magnetic resonance imaging of tumor with a self-traceable phosphorylcholine polymer. *J. Am. Chem. Soc.* **137**, 799–806 (2015).
